# ‘It’s like a personal motivator that you carried around wi’ you’: utilising self-determination theory to understand men’s experiences of using pedometers to increase physical activity in a weight management programme

**DOI:** 10.1186/s12966-017-0505-z

**Published:** 2017-05-05

**Authors:** Craig Donnachie, Sally Wyke, Nanette Mutrie, Kate Hunt

**Affiliations:** 1 MRC/CSO Social and Public Health Sciences Unit, Institute of Health and Wellbeing, University of Glasgow, 200 Renfield Street, Glasgow, G2 3QB UK; 2 School of Social and Political Sciences, Institute of Health and Wellbeing, University of Glasgow, 25-29 Bute Gardens, Glasgow, G12 8RS UK; 3 Moray House School of Education, Institute for Sport, Physical Education and Health Sciences, University of Edinburgh, 2.27 St Leonard’s Land, Edinburgh, EH8 8AQ UK

**Keywords:** Obesity, Self-monitoring, Feedback, Lifestyle intervention, Behaviour change, Qualitative methods, Men’s health

## Abstract

**Background:**

Self-monitoring using pedometers is an effective behaviour change technique to support increased physical activity (PA). However, the ways in which pedometers operate as motivational tools in adoption *and* maintenance of PA is not well understood. This paper investigates men’s experiences of pedometers as motivational tools both during and after their participation in a 12-week group-based, weight management programme for overweight/obese men, Football Fans in Training (FFIT).

**Methods:**

Semi-structured telephone interviews were conducted with 28 men, purposively sampled to include men who did and did not achieve 5% weight loss during the programme. Data were analysed thematically utilising the framework approach, using Self-Determination Theory (SDT) - namely concepts of behavioural regulation and the basic needs of relatedness, competence and autonomy - as an analytical lens.

**Results:**

During the programme, FFIT’s context and fellow participants supported relatedness and encouraged use of the pedometer. The pedometer was seen to provide tangible proof of progress, thus increasing competence for change, whilst the ability to monitor one’s own progress and take remedial action supported autonomy; these men portrayed the pedometer as an ‘ally’. However, a minority found the pedometer ‘dispiriting’ or controlling when it evidenced their inability to meet their PA targets.

After the programme, some men no longer used the device as they had fully internalised their motivations for increased PA. In contrast, others continued to use pedometers or progressed to other self-monitoring technologies because it was enjoyable and facilitated maintenance of their increased PA. However, the minority of men who experienced the pedometer as controlling no longer used it. They were less successful in achieving 5% weight loss and appeared reliant on external factors, including support from coach and group members, to maintain motivation.

**Conclusion:**

These findings show how self-monitoring using pedometers and associated goal setting supported the development of autonomous motivation for PA, during and after participation in a group-based programme. They also suggest that programmes could focus on early identification of participants who remain motivated by extrinsic factors or express negative experiences of self-monitoring tools, to offer greater support to identify the benefits of PA based on a person’s own values.

**Electronic supplementary material:**

The online version of this article (doi:10.1186/s12966-017-0505-z) contains supplementary material, which is available to authorized users.

## Background

### Facilitating behaviour change to support weight management and increased physical activity

Physical inactivity is one of the leading causes of mortality worldwide and is associated with greater risks of several non-communicable diseases [1]. Globally, around 30% of adults are insufficiently active [2]. Current UK guidelines suggest that adults should achieve a minimum of 150 min of at least moderate-intensity physical activity (PA) per week [3].

Walking is a highly accessible form of moderate-intensity PA, requires minimal skill or equipment, has a low risk of injury, and provides many physiological and psychological benefits [4–7]. Walking interventions are effective in increasing PA levels [8] although most evidence comes from research in predominantly female samples [8, 9] and there is a relative lack of research among men in PA intervention studies more generally [10, 11].

Low levels of PA are associated with increased prevalence of obesity [12]. While improving both diet and PA are critical for successful weight *loss*, increased PA levels play a vital role in *maintaining* weight loss long-term [13–15]. While much is known about the processes underpinning the adoption of PA, there is a need for greater understanding of long-term PA maintenance [16].

Self-monitoring of behaviour is one of the most effective behaviour change techniques to support weight loss and increased PA [17–19]. Recent findings suggest behaviour change techniques such as goal setting and self-monitoring might operate differently depending on whether the object is to initiate or to maintain behaviour change [18]. Specifically, there is some evidence to suggest that, whereas goal setting is important for initiation of change, self-monitoring of behaviour may be especially important not only for initiation but also for facilitating maintenance of behaviour change, particularly for weight management [18]. These findings are in line with theoretical models that distinguish between motivational and post-motivational phases of behaviour change (e.g. [20, 21]).

Pedometers offer a simple and convenient means of quantifying and self-monitoring walking, are useful as a motivational tool and associated with effective interventions for increasing PA [22, 23]. Pedometer-based interventions are effective in promoting sustained increases in PA levels over 12 months [24] and yield modest levels of weight loss [25]. Recent findings show men responded well to pedometers as part of a weight management programme and viewed them as a popular tool for self-monitoring PA [26]. However, the ways in which pedometers operate as motivational tools in the adoption and maintenance of PA are not well understood. Greater understanding of how pedometers function as behaviour change tools in motivating initiation *and* maintenance of behaviour change would assist in the refinement of PA and weight loss interventions that seek to promote long-term increases in PA.

Self-Determination Theory (SDT; [27, 28]) offers a theoretical framework for understanding motivations underpinning PA. According to this theory, there are distinct forms of motivation on a continuum ranging from *amotivation* (i.e. person lacks intent to take action), to *extrinsic motivation* (i.e. behaviour is performed to achieve an outcome independent from the behaviour itself), to *intrinsic motivation* (i.e. behaviour is performed because it is inherently enjoyable in itself). Moreover, within SDT, there are four forms of extrinsic motivation: *external regulation* (i.e. behaviour is performed in response to external pressure, such as rewards or to avoid punishment); *introjected regulation* (i.e. behaviour is performed in response to internal pressure, such as avoidance of guilt or anxiety); *identified regulation* (i.e. behaviour is motivated by the perceived value of its associated outcomes); and *integrated regulation* (i.e. behaviour is motivated not only by its valued outcomes but also because it has been assimilated with one’s beliefs and values).

Internalisation of extrinsic motivations can occur when individuals recognise the values underpinning behaviours, assimilate them within their sense of self and develop a sense of ownership over the behaviours [27]. A linear progression along the continuum through each stage of internalisation is not seen as essential [29]. Rather, an individual can adopt a particular behavioural regulation at any time depending on prior experiences or situational factors [29].

Recently, a broader conceptualisation has been established between autonomous versus controlled motivation [30]. *Autonomous motivation* is defined as the degree to which someone perceives their actions to be self-endorsed and performed with a sense of willingness, self-reflection and free choice (i.e. identified, integrated or intrinsic regulations). Conversely, *controlled motivation* refers to the extent to which one feels external pressure or coercion to perform a behaviour (i.e. external or introjected regulations) [30].

According to SDT, the quality of motivation (autonomous vs controlled) and the rate at which motivation moves from extrinsic to autonomous depends on support for ‘innate needs’ within a particular setting or social environment. These needs are *autonomy* (the need to feel volitional and the originator of one’s actions), *competence* (the need to feel optimally challenged and able to interact effectively in one’s environment) and *relatedness* (the need to feel connected or close to others and supported in one’s pursuits). When these are satisfied, individuals experience greater self-motivation, wellbeing and healthy psychological development [31]. Conversely, when they are not satisfied, the internalisation process can be forestalled; regulations remain external or only partly internalised, leading to less self-determined forms of motivation [27]. Socio-environmental conditions which support relatedness, competence and autonomy are conducive to more autonomous forms of motivation which, in turn, are associated with more adaptive behavioural and health outcomes [32].

In recent years there has been a proliferation of SDT-based research on exercise and PA [33], and on long-term weight control [34]. Accumulating evidence indicates that greater autonomous motivation is associated with long-term increases in PA/exercise behaviour [35] and successful weight control [36]. Previous studies have qualitatively explored the motivational dynamics associated with long-term PA behaviour grounded in the SDT framework and found that self-worth and internalisation of motivations were key for maintenance of PA (e.g. [37–39]). These studies were mainly conducted with women. If findings are similar in men who have taken part in a healthy lifestyle intervention, this would support wider generalisability and could help improve programmes to support long-term change.

All men in the current study had participated in a weight management and healthy living programme known as Football Fans in Training (FFIT). This analysis extends previous research on men’s experiences of using pedometers as an essential part of the walking component of FFIT during pilot deliveries of the programme [26]. A central aim of the current study was to gain greater insight into the mechanisms through which pedometers as self-monitoring tools influence men’s motivation for increased PA during *and after* taking part in the 12-week FFIT programme.

## Methods

### Setting and context for current study: football fans in training

FFIT is a gender-sensitised, weight loss and healthy living programme, delivered free of charge at professional Football Clubs by club community coaches to overweight/obese men (BMI > 28 kg/m^2^) aged 35–65. The development of FFIT is reported elsewhere [40]. In short, the FFIT programme both drew on evidence of what is most effective for weight loss [41, 42], and was devised to work with rather than against dominant constructions of masculinity, in recognition of the importance of health behaviours as an important means of ‘doing’ gender [26]. Thus FFIT was designed to appeal to men in: context (professional football clubs), content (e.g. information around the science of weight management presented simply; branded materials, such as club T-shirts) and style of delivery (e.g. coaches encourage peer-support, participative learning and positive ‘banter’ to support discussion of more sensitive issues) [43]. The FFIT programme was not based on any single theory of behaviour change [43]. Rather, it drew on a range of behaviour change techniques, including self-monitoring, implementation intentions, goal setting and review, and feedback on behaviour which have been shown to be effective in PA and healthy eating interventions [17–19]. FFIT also encourages social support and includes a focus on relapse prevention strategies. Whilst FFIT was not explicitly based on SDT in its development, components of the SDT framework were considered to provide a useful framework in understanding factors associated with weight loss maintenance at 12-month follow-up [43].

A core component of FFIT is a pedometer-based incremental walking programme based on Walking for Wellbeing in the West [4, 24]. This focuses on gradually increasing the amount of walking people perform in daily life, using weekly goals to increase their average step count. Each man is given a pedometer as a tool allowing real-time feedback on number of steps performed; to minimise programme delivery costs, and thereby maximise the potential for wider implementation post trial, a simple and cheap pedometer was provided (Silva Ex Step). The programme materials also include a diary in which participants record their progress. In week one, each man is asked to use the pedometer to record his daily step counts over the following week, to establish his baseline average as a benchmark against which to set targets over the 12-week programme. As the programme progresses and men’s physical fitness increases, participants are encouraged to incorporate other forms of PA, in addition to walking, to achieve their weekly activity goals [43]. This might include more vigorous activity but men are also given a ‘rule of thumb’ that 10 min moderate activity (such as swimming or cycling) is roughly equivalent to 1000 steps.

An evaluation of FFIT (funded by NIHR) was enabled by the provision (by Scottish Government and the Football Pools) of funding for deliveries of FFIT in 2011–2012. This randomised controlled trial (with embedded process evaluation) used an intervention (commenced FFIT within 2–3 weeks of baseline, pre-randomisation measurements) and waitlist comparison group (offered a place on FFIT commencing after 12 month trial outcome measures had been collected) design. The RCT results showed that FFIT attracted men from across the socio-economic spectrum at heightened risk of ill-health (based on baseline body mass index (mean BMI 35.3 kg/m^2^, sd 4.9) and waist circumference (mean = 118.4 cm, sd 11.7)) [44]; and showed clear differences in weight loss 12 months after baseline (primary outcome) and in PA, diet, and other secondary outcomes, all in favour of the intervention group [43, 45].

Because the funding for deliveries of FFIT in 2011–12 was for three separate deliveries across the clubs then in the Scottish Premier League, although only those participating in the first (August 2011) and last (August 2012) deliveries were required for the trial, it was considered unethical to assign some men to a 12-month waitlist comparison group (August 2012 delivery) if earlier, unfilled, places (in February 2012) were available. Hence, at the time of baseline measurement, the FFIT research team recruited sufficient men to fill places on *all* places then available for FFIT. Men were invited to baseline measurement sessions at the participating professional football clubs in Scotland, after which, 374 men were randomly allocated to the intervention (commenced FFIT in August 2011) and 374 to the waitlist comparison group (commenced FFIT in August 2012, after 12 month outcomes had been assessed, and the remaining 306 men were allocated to the February 2012 delivery, hence referred to here as the ‘non-trial’ delivery. This process ensured that only those who had expressed interest in FFIT at the time of baseline measurements could take part in any of the available deliveries; anyone expressing an interest in FFIT after the baseline measures was placed on a waiting list for potential future deliveries in 2013. It also presented opportunities for further research on FFIT, by involving men who took part in the ‘non-trial’ deliveries in FFIT, without the risk of overburdening participants in the trial.

### Design

The study reported here was a qualitative, semi-structured, telephone interview study with a subsample (*n* = 28) of FFIT participants, purposively sampled from men offered places at four of the 12 clubs which ran ‘non-trial’ deliveries of FFIT commencing February 2012. Thirty-four men (of 94 who had been assigned to February 2012 deliveries of FFIT at these four clubs), were contacted and invited to take part in a telephone interview. One refused or withdrew, another was not contactable and four did not respond when telephoned despite appointments being arranged. Interviews were conducted until data saturation had occurred. We aimed to interview roughly equal numbers of men who had and had not lost 5% or more of their body weight, a marker of successful weight loss [41, 42]—the primary focus of the programme—during their participation in the 12-week programme, as we wished to explore whether there were any differences in the accounts of these two groups. Fourteen men in the sample had lost 5% or more of their body weight, whereas 12 men had not achieved 5% weight loss at 12-weeks, and weight outcomes were missing for two men (see Table 1). All FFIT participants were made aware at the beginning of the programme what a 5% weight loss target would be for them. Ethical approval was granted by the University of Glasgow, College of Social Sciences Research Ethics Committee (CSS201020106).

**Table 1:** Participant characteristics, ordered alphabetically by pseudonym

Pseudonym	BMI Category (baseline)	Achieved at least 5% weight loss 12-weeks	% weight loss 12-weeks	Age	Marital status	SIMD	BMI (baseline)	Club ID
Alan	Obesity I	No	2.06	44	Single	5	31.3	02
Alex	Obesity III	Yes	5.94	42	Cohabiting	1	41.9	01
Andrew	Obesity II	No	3.82	41	Married	1	36.5	02
Ben	Obesity I	No	n/a^b^	36	Separated	3	34.4	04
Billy	Obesity II	Yes	7.5	52	Married	5	38.9	03
Calum	Obesity II	Yes	5.17	38	Divorced	1	39.7	01
Chris	Overweight	Yes	8.8	58	Married	5	28.5	02
Dan	Overweight	No	n/a^b^	58	Married	5	26.6	02
David	Overweight	Yes	5.74	49	Separated	3	29.1	04
Donald	Obesity III	No	n/a^b^	49	Married	2	46.0	04
Frank	Obesity II	Yes	9.67	49	Married	1	35.7	01
Gary	Obesity I	No	4.32	50	Married	5	32.0	02
George	Obesity II	Missing^a^	Missing^a^	56	Married	5	35.2	03
Gordon	Obesity I	No	1.87	40	Divorced	1	34.6	04
Grant	Obesity III	No	2.09	58	Married	1	42.0	04
James	Obesity I	No	3.5	42	Cohabiting	2	30.5	04
Jamie	Obesity I	Yes	6.07	36	Married	3	31.3	02
Jeffrey	Obesity I	Yes	9.78	53	Cohabiting	2	33.1	04
Jonathan	Obesity III	Yes	15.99	47	Married	2	43.0	03
Kevin	Obesity I	No	n/a^b^	47	Cohabiting	3	32.0	02
Martin	Obesity III	Yes	10.31	34	Married	2	42.8	02
Matthew	Obesity III	Missing^a^	Missing^a^	47	Married	3	40.4	03
Michael	Overweight	Yes	7.58	55	Married	2	27.1	01
Ross	Obesity II	No	1.92	52	Married	2	38.4	02
Ryan	Obesity III	Yes	11.52	54	Married	4	42.4	03
Steven	Obesity II	Yes	5.43	33	Married	4	36.9	01
Thomas	Overweight	No	1.65	37	Separated	3	29.1	02
Tim	Obesity I	Yes	6.86	33	Cohabiting	5	31.7	02

^a^Missing: data missing, ^b^n/a (non-applicable): did not achieve weight loss, *SIMD*: Indicator of level of affluence/deprivation of areas of residence using Scottish Index of Multiple Deprivation (Quintiles), 5 = lowest quintile of deprivation

### Data collection

Interviews were conducted some months after the completion of the programme so that post-programme experiences of lifestyle change could be addressed. The first author (CD) telephoned men from a private office, and in an email confirming the appointment, participants had been encouraged to receive the call in a setting that enabled them to speak freely, without distraction. Consent was audio-recorded. CD had met all men in person on two previous occasions at pre- and post-programme measurement sessions in the clubs. Interviews were conducted between September 2012 and February 2013, and most lasted 60 to 90 min. A topic guide included a range of questions, but all men were specifically asked about their use of the pedometer (Additional file 1; see [46] for full version). The topic guide was not explicitly designed to investigate concepts associated with SDT. The relevance of SDT-based concepts became more salient at a later stage of analysis and interpretation of the data.

### Data management and analysis

Interviews were digitally recorded onto an internal server to ensure security and optimise recording quality, and transcribed verbatim. Transcripts were checked against the original recordings for accuracy. Men were given pseudonyms and clubs were allocated identification numbers to ensure anonymisation. Nvivo software (QSR International Pty Ltd. Version 10, 2012) was used to facilitate data storage and retrieval. Data were analysed thematically utilising principles of framework analysis [47, 48], an increasingly popular method in qualitative health research [49]. Each transcript was read repeatedly and main themes identified both deductively, based on the research objectives, and inductively, based on ideas that emerged as analyses continued [47]. CD was the primary analyst.

In accordance with the framework approach, there were five main stages of analysis: familiarisation, identifying a thematic framework, indexing, charting, and mapping and interpretation [48]. First, each of the interview transcripts was read several times by CD prior to formal analysis. During this process of familiarisation, initial thoughts were noted down focusing mainly on what participants said about use of pedometers during the walking component of FFIT; SW and KH read a sample of transcripts to verify key themes. CD then initially coded to agreed broad headings including: what men said about using the pedometer *during* FFIT; what men said about using the pedometer *after* the 12-week programme; men’s references to self-regulation (e.g. self-monitoring and goal setting); motivations for PA and pedometer use; the role of others in pedometer use; and perceptions of walking/PA. Outputs from these broad codes were read and discussed by three authors (CD, SW, KH). Once the transcripts were coded to these themes the content within each theme was charted and summarised into framework matrices by CD. In this approach, each participant was assigned a row and each theme presented in a column. In some cases raw data and/or direct quotations were included in the matrix to retain sufficient context. This iterative process enabled thorough inspection of the data and identification of consistencies across the themes and individuals as well as atypical cases that were inconsistent with other men’s accounts. In line with this approach we were able to visually interrogate data across each of the frameworks as well as referring back to the original transcripts. Therefore, congruent with the ‘mapping and interpretation’ phase of the analytical process, we were able to interpret the data and relate the findings to wider theoretical explanations. To enhance rigour, selected transcripts and thematic outputs were read by co-authors (KH and SW) to allow detailed discussion of the data, coding and stages of analysis. The main themes relevant to the analyses presented here were: *The physical and interactional context as a source of motivation for using the pedometer during the 12-week programme; Using pedometers for objective feedback and self-monitoring during the 12-week programme; and Role of the pedometer in supporting behaviour change after completion of the 12-week programme*.

Having noted the range of men’s accounts of pedometer use during and after FFIT, SDT-based concepts were integrated as an interpretive lens to further inform our analysis across the three main themes. Congruence with key theoretical tenets of the SDT framework were noted, namely in relation to motivation and behavioural regulation. SDT was used as a guiding framework to understand the ways in which the pedometer influenced men’s motivations for adopting and sustaining increases in PA both during and after taking part in the 12-week FFIT programme.

The ‘One Sheet of Paper’ method [50] was employed at this stage of analysis; this requires close inspection of data coded to each theme and recording, under distinct headings, all examples of issues arising in the data, noting respondent details next to each extract. This approach allows the identification of the range of experiences under each main theme and any unexpected findings or ‘deviant cases’. Systematic comparisons were conducted between the accounts of men who achieved and did not achieve 5% weight loss post-programme across the three consolidated themes, to identify any differences in the language and responses between the two groups. The study adhered to the Consolidated Criteria for Reporting Qualitative Research guidelines [51] (see Additional file 2). Men were offered a £20 club shop voucher to thank them for participating in the interviews.

## Results

Interview respondents’ characteristics are displayed in Table 1, which shows the wide range in weight loss at 12 weeks (1.87–15.99%) amongst this purposively selected subsample. Almost all men agreed that walking was a simple form of PA that nearly anyone could do to increase their fitness and achieve health benefits. For example, Ben described walking as: “actually a very natural activity […] everybody has the opportunity to walk.” Walking was seen as very accessible as it incurred little or no financial costs and required no special equipment. The graduated walking programme enabled FFIT participants to increase their activity levels, without putting them at risk of injury or adverse health consequences or compromising their self-image. For example, Jonathan said: “I have got a problem with my right knee, […] I don’t particularly like swimming […] being the weight that I was […] prefer walking because I don’t look so stupit [stupid].”

### The physical and interactional context as a source of motivation for using the pedometer during the 12-week programme

The professional football club setting was described as an extremely important motivating factor as it provided men with an opportunity to engage with components of a weight management programme that felt ‘right’ for them. The people, including the community coaches and other men on the programme perceived as being ‘like them’, provided a combination of facilitative and supportive roles. Thus, the physical and interactional context of the programme created a motivational climate which provided optimal conditions for the men to adopt behaviour changes and engage with behaviour change strategies and tools, including the pedometer-based walking programme.

Some men spoke explicitly about the importance of being perceived by other FFIT participants as able to keep up with their PA goals; they seemed to feel accountable to other group members. For these men reaching their step-based targets, *combined with* a perceived need or desire to report back to the group, was described as particularly motivating:

*You were given, obviously, targets every week, to achieve a daily step-count. And since you had that target, it kind of spurred you on a little bit, every day or every week, to make sure you reached, or exceeded that target. And I think, again, going back to a kind of stereotypical man […] you know a bit of competition there to make sure you’re not the one in the group that doesn’t reach the weekly target. You know, you want to do well for yourself, but you also want to kind of prove to the group that you are […] doing what you’re meant to. (Thomas)*

Therefore, some men appear to have been motivated to use the pedometer and adhere to the walking programme by a desire or need to report back to the group, to avoid guilt or to support feelings of pride (in themselves as men), by being seen by men in the group as able to achieve their step-based targets and to feel connected to a group they valued. In accordance with the SDT framework, these men’s accounts are indicative of introjected regulation, whereby their behaviour had become partially internalised but remained contingent on internal rewards (e.g. feelings of pride) or avoiding punishment (e.g. feelings of guilt).

### Using pedometers for objective feedback and self-monitoring during the 12-week programme

Most men described the pedometer as a useful and valuable technology for monitoring activity. It was perceived to be easy to use, portable and non-intrusive: George described it as “so small, it’s no’ [not] uncomfortable, it’s no’ heavy, nothing like that.” Most said they enjoyed wearing the pedometer and a few described it as one of the most valuable components of the programme. For example, Chris said “I loved it. Absolutely loved it. […] I thought it was one of the best things that we got.” Some men described the first few days of wearing the pedometer as ‘strange’, but most said they soon got used to wearing it as part of their daily routine: Billy described how “after a couple o’ [of] days you automatically put it on […] it’s no’ a chore, […] it becomes part o’ you sorta thing.” Some expressed shock at how low their step count was initially. For example, Michael said “It kind of confirmed how sedentary I was […] how little exercise I was actually getting”. This ‘shocking’ realisation appeared to increase their motivation to increase their PA levels through the walking component of FFIT. Thus at the outset of the programme, the pedometer provided men with an awareness of their (in)activity levels which they felt they could not contest. The pedometer feedback therefore gave the men personally relevant information and a meaningful rationale for increasing their activity levels, whereas the graduated walking programme provided them with an opportunity to do so. Hence, for the majority of men the pedometer feedback was perceived as motivating as it facilitated self-initiated (i.e. autonomous) reasons for increased PA, consistent with the SDT framework.

Having gained a clearer understanding of their baseline activity levels, most men described the importance of setting goals each week to increase their activity levels, particularly during the first few weeks of FFIT. Respondents described how instantaneous feedback from the pedometer throughout the day helped them to achieve their PA goals by enabling them to determine precisely how far they were from their goal. Their accounts were redolent with references to self-regulation, self-monitoring and goal setting. For example, several described a strong drive to achieve their PA goals, especially during the 12-week programme:

*I mean it actually showed you the lack of exercise you’re doing because obviously it counted the steps you were doing. So, let’s say you had […] five thousand steps to do that week, right? a) it gi’d [gave] you how much steps you were walking normally. And, b) because you were doing it you said “No, I can beat that, I can do better than that”. You walked long distances round rather instead of taking the short direct routes… I suppose it’s like a personal motivator that you carried around wi’ [with] you. (Matthew)*

Most men used language that implied that self-monitoring with the pedometer supported feelings of competence and mastery, providing optimal challenge by gradually increasing their step-based goals at their own pace, which, in turn, was described as being ‘fun’/’enjoyable’ in itself. The feedback was often described as ‘*proof’* or *evidence* not just of whether they had successfully increased their PA levels, but that this was a behaviour that they could change and was surprisingly achievable:

*it gave me the proof, if you want, that I could, without any great effort actually, just increase […] the numbers of steps […] if I hadn’t quite managed to get as many steps as I’d wanted, yeah it gave me the perfect sort of push. (Dan)*

A few men emphasised the importance of their PA behaviour being self-initiated and under their own volition, and not feeling coerced to increase their activity: “You’re not being forced to do it, you’re walking at your own speed” (Andrew). This was again facilitated by the pedometer, which was described as being akin to a ‘gauge’: “like a speedometer […] the information that you got oot [out] o’ it was self-driven” (Billy).

In explaining how the pedometer helped to motivate men to remain committed to achieving their activity-based goals and continue to self-monitor their activity levels, some used language which personified the pedometer:

*it was almost like my conscience, you can’t count steps, you maybe think that you’ve done a lot of walking, but unless you’ve actually got the device there telling you what you’ve done then you don’t know for sure and you could let it slip, […] you’ve only got your own mind telling you to do things […] if you’ve not got something concrete to prove that you’ve been active […] in black and white and dark grey and light grey [colours of the pedometer screen], sitting there telling ye [you]. (Gary)*

Therefore, several men portrayed the device as an *ally* or *facilitator* that helped them to keep a track of, and achieve, their goals. This in turn helped to internalise self-regulatory habits (i.e. self-monitoring) into daily life. The way in which some used language to personify the pedometer, is also consistent with introjected regulation.

However, a few men portrayed the pedometer as a tyrannical presence in their lives if, as the weeks progressed, they began to find their step-based goals too much of a challenge. It is important to reiterate here that the walking programme was designed so that once men achieved a certain level of fitness and/or minimum of 4500 more steps per day over their baseline step count, that this was seen as an adequate and health enhancing level which they should then aim to maintain. They were then encouraged to incorporate other forms of PA which suited them, and to equate 10 min of moderate-intensity PA with an additional 1000 steps to provide variety to the mode of activity by which they accumulated steps. It appears that this message had not been communicated clearly enough or assimilated by a minority of men who consequently self-imposed a very challenging step count goal:

*I knew that if I do 8000 steps a day they [would] want me [to do] five hundred and a thousand […] all of a sudden I knew that it would be 20,000 steps a day […] I don’t think it’s sustainable. So […] at the start I was going “Right, well keep it going, if I can get tae 10,000 and no anymore than 15,000, I think that’s sustainable.” […] But it was […] things like 18,000 steps a day I had to do. And then I was realising, “Here I cannae make 18,000 steps a day.” And I was coming home and I was looking at my step counter [pedometer] like, “Aww maybe 3000 steps short of my – my target.” And I was running on the spot to make up my 3000 steps and it might take me fifteen minutes, twenty minutes […] it was like a governor, you were ruled by this pedometer.*

Therefore, for a minority of men, if their step-based targets increased beyond a level they perceived to be achievable, the pedometer was constructed less as a positive tool; rather, it became a tyranny and an oppressive/controlling device which undermined their autonomy.

### Role of the pedometer in supporting behaviour change after completion of the 12-week programme

Several men reported continuing to use the pedometer to monitor their activity levels regularly or intermittently after they had completed the 12-week programme. Some viewed wearing the pedometer as an important means of continuing surveillance of their PA to ensure they were maintaining their activity levels:

*CD: Do you still find the pedometer helpful?*

*Ben: Yes but not in the same way, whereas previously the pedometer […] was a motivating thing. It was the stick that I controlled myself and motivated myself to go and do it, whereas now, yeah, it’s a useful function as a tool so when I am out walking it’s a way of judging how much walking I am actually doing. (Ben)*

The majority of these men still enjoyed wearing the pedometer as it reinforced feelings of achievement in maintaining their increased PA. However, these men also discussed several benefits associated with walking and/or becoming more active which prompted them to continue their activity levels (e.g. feeling healthier):

*I’m in the culture noo [now] […] I’m still doing aboot ten mile a day. […] It definitely helps, tae [to] have a pedometer on and going out and enjoying a walk […] knowing that it’s there and it’s recording every step. I think if you didnae’ have one you wouldnae’ feel as motivated, you know? I think it’s a good motivation tool […], you can see what you’re actually achieving […] it’s good for your confidence […] it definitely makes you feel better […] I’m thoroughly enjoying it […] you look forward tae [to] going for it now […] I definitely feel a lot healthier now than I did last year before I started this programme […] being oot [out] in the fresh air and meeting people […] it’s great for your wellbeing. (David)*

One man articulated how the pedometer also served as a symbolic representation of his broader intentions to maintain a healthier lifestyle, which went beyond providing a literal representation of his activity levels:

*It’s also something, a physical thing, to represent some of the changes that I’ve made […] even if I don’t look at the steps it reminds me that I’m, I’ve got a different view about exercise now […]. It’s almost like a totem in that respect. […] it’s like the badge; it’s like the certificate really that you can carry about pretty discretely without people really noticing to remind you of the success that you’ve had […]. So it was a big part of, I suppose, the daily motivation to do something and I still wear it and that’s been months now since I finished the programme, I still use it. (Frank)*

For other men the pedometer was as a first step towards adopting more sophisticated forms of ‘*self-tracking’* technologies (e.g. smart phone applications, mobile devices) to monitor their activity levels following the programme:

*I quite like my technology, so I use an app called Map My Run on my smartphone, and that, you know, that uses GPS […]. You know, it literally tracks you, exactly where you are, how fast you’re going, how much …of an incline […] it gives you a kind of full readout of your workout […] it’s just an extension of the likes of a pedometer. […] it allows you to kind of map that, and your progress, week on week. You’re going a bit further, you’re going a bit faster […] where the programme really helped was […] getting you into a cycle of, you know, recording and monitoring and actually seeing progress. (Tim)*

Despite the perceived benefits of continuing an active lifestyle beyond FFIT, these men remained focused on (and enjoyed using) external quantification and surveillance technologies for maintaining increased PA. Nevertheless, it was clear that these men perceived the tangible evidence provided by self-monitoring as important and satisfying, and as a result they continued to enjoy active lifestyles. While the majority of these men’s accounts were indicative of adaptive forms of motivational regulation (e.g. intrinsic and identified regulations) they also used language demonstrative of controlling forms of behavioural regulation (i.e. introjected regulation).

In contrast, several men no longer felt the need or desire to use the pedometer and/or other self-monitoring technologies once the programme had ceased. These men perceived themselves as having more options in relation to the kinds of activity they now felt capable of. These pursuits were described as inherently enjoyable, exciting and provided them with a new-found sense of autonomy. Some explicitly said it was self-monitoring their activity with the pedometer that was the catalyst that enabled this longer term change, not just in their activity levels but also their sense of self:

*At first I was walking […] by the end of the course like I was, I was running […]. [B]y being able to measure and try and beat the previous day’s measurements […] that was just started with using the pedometer and walking as part of the programme. Like without that I wouldn’t have, I wouldn’t have done a marathon […] my physical fitness improved and sort of spurred me on to do more and more. And actually like came to really, really enjoy it. And it’s became a sort of passion of mine. […] it’s just something that’s y’know I’ve sort o’ really, really enjoy. And feel more able to do it now […] I’m a lot fitter now so it’s less of a, a chore. And sort of the more I do it the easier it becomes, the better I become at it, the more I want to do it. […] I mean I just love it. I’ve done maybe half a dozen half marathons and different runs and cycling sort o’ [of] fair distances. […] it’s absolutely brilliant. And I, I feel so much fitter and healthier. (Jeffrey)*

Many described a new or renewed sense of self and an increased sense of vitality, both through their physical bodies (e.g. weight loss, feeling healthier and fitter) and psychologically (e.g. greater confidence and wellbeing). These experiential benefits had emerged as being some of the most salient factors underpinning the men’s reasons for their ongoing increased PA. Hence, the role of the pedometer as a motivational tool had for them become obsolete or redundant beyond the programme:

*I started walking, as suggested on the programme, […] I had the step, the pedometer on, you know you noticed the amount of steps you were doing, you increased them […] and then the impact […] starting to see the pounds coming down on the scales made a huge difference […] the more you exercise you get the good feeling endorphins etc. […] it makes you feel better physically, it makes you feel better mentally. […] I would have to say for the duration of the programme, I actually found it very useful, to be perfectly honest, I don’t use it at all now, but then I’m going training two nights, you know Monday and Wednesday […] so I know I’m getting […] the exercise that I need. (Michael)*

These men’s accounts suggest they had internalised regulations for increased PA. This was illustrated by the fact they had redefined themselves by integrating newly (re)discovered activities which they valued into their daily lives. Although the use of the pedometer was viewed as instrumental in the early days of the programme, they no longer needed it. Since doing FFIT, the activity they did had become a part of who they were, their revised identities, and they really enjoyed it. Congruent with the SDT framework, these men’s accounts were illustrative of autonomous motivation for continued PA.

However, some described a lack of interest in using the pedometer and/or maintaining their activity levels once the 12-week programme had ended. These men were more likely to report negative experiences of using the pedometer *during* the programme and to discuss finding it more difficult to motivate themselves to sustain their activity levels post-programme:

*I’ve got other things to do, other aspects of this word “family life” therefore it’s very difficult then to sort of say, “I need to go out and do ten thousand steps,” […] you’re chained to a desk […] the amount of walking about you can actually physically do during the course of a day or a working day isn’t very high […]. I found it [the pedometer] a wee bit dispiriting if you like. […] It started off as maybe being five thousand steps or something like that and before you know it it’s meant to be twelve and fourteen […] I don’t really use the pedometer […]. I try and keep away from the pedometer if you like. (Alan)*

These men also used language to indicate feelings of self-worth contingent on their achievement of their PA targets during the programme. They reported feeling ‘disappointed’ when they were unable to fulfil their step targets and were less likely to continue wearing the pedometer post-programme:

*And you would feel good about yourself, you say if you got more than ten thousand steps in a day or whatever. And that also sometimes you maybe feel a bit disappointed if you didnae achieve the number of steps that you wanted to achieve. […] I’m doing significantly less steps now than I was when I was wearing it [the pedometer] because I’ve no’ got that, you know, there’s no’ a target there that I’m trying to achieve. (James)*

The majority of these men were predominantly motivated externally whilst on the programme, in respect of reporting their activity level back to the group instead of increasing their activity levels mainly for themselves. Thus, for these men the social support they gained whilst actively on the programme appeared to be more important in influencing their motivation for increased PA than having a technology for self-monitoring *per se*:

*You don’t maybe go out and exercise the way that you thought you might go out and exercise. I’ve just allowed that to drift. I don’t seem to find the time that I should […] I mean that’s obviously for me to address in my own time […] but I don’t think there was very much aftercare […] it feels it just you know the thirteen week programme came to an end, it was almost like a guillotine. (Donald)*

These men explained their lack of success by recounting problems they faced, which they largely constructed as beyond their control. These men were also more likely to discuss the centrality of peer support from the group or coaches for their continued motivation during the programme; the pedometer for them appeared to have been useful as a motivational tool *only* within the context of the programme. They predominantly used language suggestive of controlling forms of motivational regulation (i.e. external and introjected regulations). Further, despite being sufficiently motivated to sign up for the 12-week programme, some used language to suggest they were moving towards amotivation.

## Discussion

This analysis of men’s experiences of using pedometers within a weight management programme provides further insight into the processes through which self-monitoring tools influence men’s motivation for sustained behaviour change, and hence early indicators of which participants may need further support to adopt behavioural change. The findings illustrate how pedometers as self-monitoring tools can be used to facilitate adaptive forms of motivation for sustaining increased PA. Men who used the pedometer initially but no longer used it post-programme whilst maintaining sustained increases in their PA, appeared to have developed autonomous motivation. Hence, they no longer required self-monitoring technologies to sustain their PA. Even within the later stages of the 12-week programme, self-initiated reasons for performing PA had become more salient to these men. Nonetheless, the pedometer was cited as a vital catalyst and motivational tool in the early phases of the programme. Thus, from an SDT viewpoint, these men’s motivations appeared to be fully self-determined and therefore more likely to endure long-term; this relationship with self-determination has been shown in other research (e.g. [33]).

Conversely, some men who reported sustained increases in PA continued to use the pedometer and/or alternative self-monitoring technologies post-programme. For them, quantification of PA was viewed as an important means of ensuring sustained PA, and they described their continued use of self-monitoring technologies as something they enjoyed and even a symbol of a newly integrated repertoire of behaviours. Within SDT, this form of motivation may be viewed as somewhat extrinsic, although these findings suggest it may not be important as they continued to maintain increased PA.

However, for a small minority, the pedometer was experienced as ‘dispiriting’ or ‘controlling’ when it evidenced their inability to achieve their PA targets, hence undermining their sense of competence. These men were unlikely to report using it or other self-monitoring tools once the 12-week programme had ceased, and more likely to indicate reliance on external factors, such as the peer support from the weekly sessions. These were all men who had not succeeded in losing weight whilst on the programme. They tended: to view their weekly step targets as being externally sanctioned rather than initiated by themselves; to display predominantly controlled motivation; to appear more likely to adopt a more rigid and inflexible approach to goal setting; and to describe the pedometer in a negative light, often using personifying terms (the ‘Governor’). This contrasts with earlier research on men, who described the pedometer only in positive ways [26].

This finding clearly illustrates a key strength of this research, namely using purposive sampling so that we could systematically compare the accounts of men who had, and had not, lost at least 5% of their baseline weight (a central focus of the FFIT programme). Our systematic comparison revealed that all men who appeared to have developed autonomous motivation as well as the majority of men who demonstrated partially internalised motivation, had successfully embedded lifestyle changes which resulted in weight loss. However, men who no longer used the pedometer and predominantly used language consistent with less adaptive forms of motivation were not as successful in losing weight during the programme. These results suggest that these men can be identified and potentially offered additional support to succeed in their goals early on in the programme.

In SDT, support and consequent satisfaction of the ‘basic needs’ for autonomy, competence and relatedness, encourages more autonomous motivation [32]. Men’s motivation to utilise the pedometer during the 12-week programme was influenced by fulfilment of these basic needs. The social and interactional context was congruent with men’s identities and promoted *relatedness*: the enhanced physical and symbolic proximity to the professional football club gained over the 12-week programme [26] inspired men to embrace the pedometer-based walking programme; and being with other participants ‘like them’, encouraged by community coaches who played supportive and facilitative roles further satisfied relatedness. These findings are consistent with previous research on FFIT (i.e. [26, 40, 43, 44, 52]) and with an SDT perspective, which posits that people are more likely to engage initially in an externally prompted activity which they view as being endorsed by a reference group (e.g. a peer group, society or culture) which they feel, or desire to feel, connected to [29]. For most men self-monitoring and feedback from the pedometer provided ‘proof’ of success in achieving graduated PA goals, enhancing feelings of *competence* in making key behavioural changes. The ability to self-monitor their step count throughout the day and to decide when and how to incorporate further steps to reach their targets, promoted men’s sense of *autonomy*, with the pedometer often constructed as an ‘ally’ in this process. In accordance with the SDT model of behaviour change [53], satisfaction of the need for autonomy is crucial for autonomous motivation and is therefore essential in both the adoption and maintenance of behaviour change.

These findings build on recent work that has qualitatively explored motivational processes in relation to behaviour change which have applied the SDT framework (e.g. [38, 39]). These studies also demonstrate the importance of ‘need’ satisfaction in facilitating autonomous forms of motivation, which are more likely to be sustained. Sabiston et al. [39] found that participants displaying feelings of competence, social connections and personal control during a 12-week dragon boat exercise programme, continued PA after the intervention. Kinnafick et al. [38] reported that participants demonstrated autonomous motivation for PA during a workplace walking intervention; some participants displayed identified regulation, such as recognising the health benefits of PA or enjoyment of walking itself, consistent with men’s accounts in the current study. They also reported that support from others was important in the adoption and maintenance of PA during the intervention, including among participants who did not adhere to PA recommendations after taking part in the intervention. Findings from quantitative research also indicate that reliance on support from others, without experiencing autonomy, is associated with less autonomous forms of motivation [54]. Our findings are also consistent with a recent qualitative study that used SDT-inspired concepts to investigate pedometer use among a sample of male and female patients taking part in a cardiac tele-rehabilitation programme [55]. Thorup and colleagues found that pedometer use enhanced patients’ feelings of autonomy, competence and relatedness. The authors concluded that pedometers promoted motivation for walking among the majority of patients, although not all aspects of their motivation appeared autonomous.

From an SDT-based perspective, there is growing empirical support for the notion that endorsement of intrinsic goals (e.g. becoming healthy or physically fit) rather than extrinsic goals (e.g. enhancement of appearance or image) for being physically active, have been positively associated with greater psychological need satisfaction, positive psychological outcomes and increased PA [56]. Our findings are consistent with a recent study that qualitatively investigated exercisers’ experiences of intrinsic and extrinsic goals [57]. Sebire and colleagues reported that participants pursuing mainly extrinsic goals were more likely to adopt a rigid approach to exercise behaviour and to focus on specific markers of goal progress. Our findings are also consistent with previous suggestions that extrinsic goals and set endpoints might facilitate a more rigid and narrowly focused approach to behavioural engagement [58]. Sebire and colleagues also found that achievement of extrinsic goals promoted controlled motivation to sustain one’s achievements. Conversely, they reported that participants pursuing predominantly intrinsic goals were more likely to adopt a flexible and long-term approach to exercise behaviour and were more likely to volitionally pursue additional challenges once they had experienced goal achievement. Moreover, intrinsic goals appeared to facilitate more autonomous motivation for engagement consistent with recent quantitative research [59]. Our findings indicate potential overlap between SDT-related concepts of goal contents and motivational regulation, particularly in relation to rigid and flexible goal setting. Future qualitative research could be utilised to further investigate the relationship between goal contents, behavioural regulations and need satisfaction among participants within the context of PA behaviour change interventions.

A practical application of these findings may be the early identification of participants who are more likely to need additional support to achieve sustainable changes, and sensitisation to these in the training of the deliverers of interventions like FFIT. Identifying those whose motivations for increasing PA are principally driven by extrinsic factors (e.g. comparison with others) or those who express negative experiences of self-monitoring tools, may suggest a need for additional focus on instilling autonomous motives (e.g. value and enjoyment).

It is important to add that there are various plausible mechanisms as to why self-monitoring of PA alongside graduated goal setting was experienced as unhelpful by a few men. First, some men appeared to have misinterpreted recommendations for a gradual increase to 4500 steps per day above baseline levels on at least 5 days of the week. Additionally, for some men, particularly those with high step counts at baseline, introducing a greater emphasis on alternative forms of PA at an earlier stage of the programme may have been beneficial and could suggest a need for clearer guidance on how to self-monitor other forms of PA. These findings emphasise the continuing importance of careful review of PA goals on a weekly basis.

The study had a number of strengths. For example, our purposive sampling strategy allowed us to compare and contrast the perspectives of men who had and had not been successful in achieving their 5% weight-loss target whilst on the programme, and so to identify from some men’s accounts potential early identifiers of relatively poor outcomes, as described above. However, the study also had a number of limitations important to consider. The men’s accounts in the study were retrospective, to capture their experiences of pedometer use both during and after taking part in FFIT. In some cases several months had passed since they had completed the programme and hence some of their accounts may have been subject to distortion or recall bias. Future research utilising a prospective design to investigate the dynamic processes underpinning motivation (i.e. behavioural regulation) and use of self-monitoring tools may be enlightening. Additionally, it is important to note that despite being encouraged to express their opinions freely at all the times, it is possible that some men may have felt prohibited from providing more critical views about the pedometer and the FFIT programme in general.

## Conclusion

Overall, these findings show that for the majority of men self-monitoring using pedometers and associated goal setting during the programme supported the development of autonomous motivation for PA by satisfying basic needs for autonomy, competence and relatedness. For some the device was no longer used after the programme as they had fully internalised their motivations for increased PA. In contrast, others continued to use the pedometer or had progressed to other self-monitoring technologies because it was enjoyable and facilitated PA maintenance. Both of these groups were successful in losing weight whilst on the programme and sustained lifestyle changes. However, a minority of men experienced the pedometer as controlling, undermining their needs for autonomy and competence. These were men who no longer used the pedometer post-programme and during the programme reported greater reliance on external factors, including support from coach and group members. These findings suggest that programmes could focus on early identification of participants who are principally driven by extrinsic factors or those who express negative experiences of self-monitoring tools and offer greater support to identify the importance of PA based on a person’s own values.

## Additional files

Additional file 1: Interview Topic Guide. (DOCX 14 kb)

Additional file 2: Adherence to items outlined in the Consolidated Criteria for Reporting Qualitative Studies Checklist. (DOCX 18 kb)

## Abbreviations

BMI: Body mass index
FFIT: Football fans in training
PA: Physical activity
SDT: Self-determination theory
