# Supplementary material for: ‘It’s like a personal motivator that you carried around wi’ you’: utilising self-determination theory to understand men’s experiences of using pedometers to increase physical activity in a weight management programme
Source: Int J Behav Nutr Phys Act. 2017 May 5;14:61. doi: 10.1186/s12966-017-0505-z (PMC5420087; doi:10.1186/s12966-017-0505-z)
Supplement: Supplementary file 1 — Interview Topic Guide. (DOCX 14 kb) [file 12966_2017_505_MOESM1_ESM.docx]

| **Additional file 1**  **Interview Topic Guide**   - As part of the physical activity component of the programme you were given a pedometer to help you keep track of your physical activity levels. I am interested in hearing about your experience of wearing the pedometer, both during and after completion of the programme. - Tell me what it was like wearing the pedometer? - Overall, how useful did you find the pedometer as a means of increasing your activity levels? In what ways did it help? - Did wearing the pedometer ever cause you any problems? If yes, can you tell me a bit more about that? Prompt, for any negative factors or barriers to using the pedometer if not mentioned - People often say that walking is a good way for someone to increase their physical activity levels. In what ways do you agree, or disagree with this? - Since you completed the programme, have you managed to stick your physical activity goals/recommendations? - Do you still find the pedometer helpful? (if yes, probe for how it is still being used; if no, probe for when/why pedometer is no longer used) |
| --- |
