# Supplementary material for: ‘It’s like a personal motivator that you carried around wi’ you’: utilising self-determination theory to understand men’s experiences of using pedometers to increase physical activity in a weight management programme
Source: Int J Behav Nutr Phys Act. 2017 May 5;14:61. doi: 10.1186/s12966-017-0505-z (PMC5420087; doi:10.1186/s12966-017-0505-z)
Supplement: Supplementary file 2 — Adherence to items outlined in the Consolidated Criteria for Reporting Qualitative Studies Checklist. (DOCX 18 kb) [file 12966_2017_505_MOESM2_ESM.docx]

| **Additional file 2**  **Adherence to items outlined in the Consolidated Criteria for Reporting Qualitative Studies Checklist** | |
| --- | --- |
| **Domain 1: Research team and reflexivity** |  |
| *Personal Characteristics* |  |
| 1.Interviewer/facilitator | CD (lead author and primary analyst) conducted all of the interviews |
| 2. Credentials | PhD, MSc, BA (Hons) |
| 3. Occupation | Research Assistant |
| 4. Gender | Male |
| 5. Experience and training | CD has a PhD and MSc and has received substantial training on qualitative methods (e.g. attended training courses delivered by NatCen Social Research and Social Research Association Scotland). CD has also worked on various projects involving qualitative methods both in his current post-doctoral role and in previous (pre-doctoral) employment in a research agency. In addition, CD has taught qualitative methods to postgraduate students |
| *Relationship with participants* |  |
| 6. Relationship established | CD had previously met each man during pre-programme measurement sessions, and had established a personal connection that was vital in building rapport with each respondent before conducting the telephone interviews |
| 7. Participant knowledge of the interviewer | Having already met face-to-face during the baseline measurement assessments, respondents were aware of CD’s status as a PhD research student who was investigating their experiences of taking part in a weight management programme (FFIT) as part of his PhD project. CD clearly positioned himself as a student researcher in order to appear as a ‘non expert’ whilst conducting the interviews |
| 8. Interviewer characteristics | CD is a white, heterosexual male from a middle class background, with a slim to medium build. When conducting these interviews he was in his late twenties and therefore considerably younger than nearly all of the men in the study. CD had recently completed a MSc in health psychology prior to commencing his PhD and had a specific interest in theoretical understandings of health behaviour change. It is therefore possible that this led to a more specific focus on particular content that emerged during the interviews. However, CD remained cautious not to provide too much information if the respondents asked for his own insight on matters relating to weight management, PA, diet or the FFIT programme, and instead encouraged them to discuss and reflect on their own experiences |
| **Domain 2: Study design** |  |
| *Theoretical framework* |  |
| 9. Methodological orientation and theory | The data were analysed thematically utilising the principles of framework analysis. The framework approach is not positioned alongside a specific epistemological, theoretical or philosophical approach and thus offers a flexible tool for researchers to identify commonalities and differences in qualitative data to generate themes |
| *Participant selection* |  |
| 10. Sampling | Purposive sampling was utilised to gain perspectives from men attending the FFIT programme at four clubs. The specific purpose was to understand how overweight and obese men used pedometers as motivational tools during and after taking in the 12-week FFIT programme, and to explore whether their accounts varied between different groups of men. Therefore, we sampled to achieve roughly equal numbers of men who had and had not lost 5% or more of their baseline weight during their participation in the 12-week programme. The threshold of 5% weight loss was selected as a marker of successful maintenance due to the significant health benefits associated with achieving a 5% reduction in body weight |
| 11. Method of approach | Respondents were contacted by telephone and asked if they would be willing to be interviewed. All respondents had received an information sheet about the study previously and were aware that they might be contacted after the 12-week programme had ended and asked if they would be willing to take part in an interview over the telephone |
| 12. Sample size | 28 men took part in the qualitative study |
| 13. Non-participation | Thirty four men were contacted and invited to take part in a telephone interview, n=1 refused/withdrew, n=1 not contactable, n=4 appointments made but did not respond when telephoned |
| *Setting* |  |
| 14. Setting of data collection | CD conducted all of the semi-structured telephone interviews within a university setting and was seated in a quiet and private room to facilitate optimal data collection, concentration and confidentiality, and to allow the interview to proceed with no distractions or audible disturbance at his end of the call. This was also important in trying to create optimal conditions for recording the interview. The interviews were conducted at a time that was most suitable for each of the respondents and therefore the majority of interviews were conducted in the evenings or at the weekend |
| 15. Presence of non-participants | The majority of respondents requested to be interviewed at home and therefore in some instances family members may have been present at the time of the interview. However, respondents were encouraged to receive the call in a setting that allowed them privacy and to speak freely without distraction |
| 16. Description of sample | Participant baseline characteristics of the interview respondents are provided in Table 1 |
| *Data collection* |  |
| 17. Interview guide | The interview guide was used during each of the interviews to ensure that similar questions were addressed during each interview, focusing on issues consistent with my research aims (see Additional file 1). However, the interview schedule served only as a general guide and the men often discussed other issues that were not covered in the guide |
| 18. Repeat interviews | No repeat interviews were conducted as part of the study |
| 19. Audio/visual recording | Each of the interviews was recorded electronically over the telephone directly onto an internal server to optimise quality of the digital interviews and to ensure security |
| 20. Field notes | Brief field notes and reflections were noted by CD following each interview |
| 21. Duration | The duration of the interviews varied considerably in length between approximately 40 and 120 minutes, with the majority of interviews lasting between 60 and 90 minutes |
| 22. Data saturation | Interviews were conducted until saturation occurred, that is, no new issues arose during the interviews that merited further data collection or would have provided greater understanding of phenomena under investigation |
| 23. Transcripts returned | After each telephone interview a copy of the digital recording was sent to an independent, approved, transcription company via a secure file sharing system. The interviews were transcribed verbatim and returned by secure links. CD checked each of the transcripts against the original digital recordings for accuracy and any amendments or changes implemented where necessary. CD ensured that any identifying information was anonymised |
| **Domain 3: Analysis and findings** |  |
| *Data analysis* |  |
| 24. Number of coders | CD developed the coding frame and coded all of the data. However, to add rigour to these processes, selected transcripts were read by KH and SW. Regular meetings were held to facilitate detailed discussion of the data and resolve any discrepancies or issues arising during the coding process |
| 25. Description of the coding tree | We initially coded to very broad headings in relation to: what men said about using the pedometer during FFIT; what men said about using the pedometer after the 12-week programme; men’s references to self-regulation (e.g. self-monitoring and goal setting); motivations for PA and pedometer use; the role of others in pedometer use; and perceptions of walking/PA |
| 26. Derivation of themes | Consistent with the framework approach, themes were derived both deductively (i.e. from the aims and objectives of the study) and inductively (i.e. from the men’s accounts) |
| 27. Software | Each of the interview transcripts was saved in Microsoft Word and imported into NVivo qualitative data analysis software (QSR International Pty Ltd. Version 10, 2012). Framework matrices for each broad theme were created using Microsoft Excel (2007) |
| 28. Participant checking | Participant checking was not utilised within the context of the study. We interviewed both men who were and were not successful in achieving weight loss and making behaviour changes after taking part in a weight management programme. For some men, negative emotions emerged during the interviews and were often suffused with issues around body image and prior experiences of being overweight or obese. Representing the accounts to the less successful men may have the potential to invoke negative emotions. |
